# Species of Cryptosporidia Causing Subclinical Infection Associated With Growth Faltering in Rural and Urban Bangladesh: A Birth Cohort Study

**DOI:** 10.1093/cid/ciy310

**Published:** 2018-04-20

**Authors:** Kevin L Steiner, Shahnawaz Ahmed, Carol A Gilchrist, Cecelia Burkey, Heather Cook, Jennie Z Ma, Poonum S Korpe, Emtiaz Ahmed, Masud Alam, Mamun Kabir, Fahmida Tofail, Tahmeed Ahmed, Rashidul Haque, William A Petri, Abu S G Faruque

**Affiliations:** 1 Department of Medicine, Division of Infectious Diseases and International Health, University of Virginia, Charlottesville; 2 International Centre for Diarrhoeal Disease Research, Dhaka, Bangladesh; 3 Department of Statistics, Charlottesville; 4 Division of Biostatistics, Department of Public Health Sciences, School of Medicine, University of Virginia, Charlottesville; 5 Department of Epidemiology, Johns Hopkins Bloomberg School of Public Health, Baltimore, Maryland

**Keywords:** cryptosporidiosis, species, growth faltering, birth cohort, Bangladesh

## Abstract

**Background:**

Cryptosporidiosis is a major cause of childhood diarrhea in low- and middle-income countries and has been linked to impairment of child growth. This study investigated the burden of cryptosporidiosis and its impact on child growth in both a rural and an urban site in Bangladesh.

**Methods:**

Pregnant women in the second trimester were identified at 2 sites in Bangladesh, 1 urban and 1 rural. Their offspring were enrolled at birth into the study (urban, n = 250; rural, n = 258). For 2 years, the children were actively monitored for diarrhea and anthropometric measurements were obtained every 3 months. Stool samples were collected monthly and during diarrheal episodes with *Cryptosporidium* infection and causative species determined by quantitative polymerase chain reaction assays.

**Results:**

*Cryptosporidium* infections were common at both sites and mostly subclinical. In the urban site, 161 (64%) children were infected and 65 (26%) had ≥2 infections. In the rural site, 114 (44%) were infected and 24 (9%) had multiple infections. Adjusted for potential confounders, cryptosporidiosis was associated with a significantly greater drop in the length-for-age *z* score (LAZ) at 24 months from LAZ at enrollment (Δ-LAZ), an effect greatest in the children with multiple episodes of cryptosporidiosis. The most common species in Mirpur was *Cryptosporidium hominis*, whereas *Cryptosporidium meleagridis* predominated in Mirzapur.

**Conclusions:**

Cryptosporidiosis is common in early childhood and associated with early growth faltering in Bangladeshi children. Predominant *Cryptosporidium* species differed between the 2 sites, suggesting different exposures or modes of transmission but similar consequences for child growth.

**Clinical Trials Registration:**

NCT02764918.

## Teaser

This birth cohort study describes the early childhood burden of cryptosporidiosis in rural and urban Bangladesh. Important findings include an association of repeated and subclinical cryptosporidiosis with growth faltering, and disparate predominant *Cryptosporidium* species in the urban vs rural site.

Diarrhea remains a leading global cause of morbidity and mortality in children <5 years of age [1]. Two recent multicenter studies demonstrated the importance of cryptosporidiosis, caused by an intracellular protozoan parasite, as a leading cause of childhood diarrheal disease [2, 3]. Many species of *Cryptosporidium* can infect humans; however, *Cryptosporidium hominis* and *Cryptosporidium parvum* are typically considered the most common [4–6]. Clinical manifestations of cryptosporidiosis are variable as symptomatic diarrheal disease occurs in only a subset of cases while most remain subclinical [7–9]. Early cryptosporidiosis, whether symptomatic or subclinical, has been associated with impaired growth and cognitive development [7, 10, 11].

Current understanding of human cryptosporidiosis is largely based upon diagnosis with overt diarrhea; yet given the associated long-term sequelae of even subclinical cryptosporidiosis, a better epidemiologic understanding in various settings is needed. In this active surveillance study, we describe the natural history of cryptosporidiosis over the first 2 years of life in 2 sites in Bangladesh, 1 urban and 1 rural. We characterize the burden of cryptosporidiosis and, using regression modeling, estimate the impact of *Cryptosporidium* infection on growth faltering. Furthermore, we describe a marked and unexpected difference in causative species, with *C. hominis* most common in the urban site compared with *Cryptosporidium meleagridis* in the rural site.

## METHODS

### Study Sites, Enrollment, and Surveillance

Prospective longitudinal birth cohorts (“Cryptosporidiosis and Enteropathogens in Bangladesh”; ClinicalTrials.gov identifier NCT02764918) were established at 2 sites in Bangladesh. Mirpur, Dhaka is a relatively poor, urban neighborhood [12, 13], and Mirzapur is a rural subdistrict located 60 km northwest of Dhaka, as previously described [14]. Further site descriptions are provided in Table 1 and the Supplementary Methods.

**Table 1.** Comparison of Demographics, Birth Anthropometry, and Socioeconomic Indicators of Enrolled Children

Characteristic	Mirpur (Urban)				Mirzapur (Rural)			
	No Crypto (n = 89)^a^	Any Crypto (n = 161)^a,b^	Diarrheal Crypto (n = 51)^b,c^	Subclinical Crypto (n = 110)^b,c^	No Crypto (n = 144)^a^	Any Crypto (n = 114)^a,d^	Diarrheal Crypto (n = 3)^c,d^	Subclinical Crypto (n = 111)^c,d^
Female sex	50 (56)	92 (57)	29 (57)	63 (57)	65 (45)	52 (46)	2 (67)	50 (45)
Household income, BDT^e^, median (IQR)	14000 (10000–21500)	12000 (10000–19500)	12000 (10000–18000)	12000 (9000-20000)	15000 (10000–28750)	15000 (10000–25250)	35000 (12000–37000)	15000 (10000–25000)
No maternal education	14 (16)	38 (24)	11 (22)	27 (25)	5 (4)	3 (3)	0 (0)	3 (3)
Mean maternal BMI, kg/m^2^ (SD)	22.8 (3.5)	23.0 (3.7)	23.0 (3.3)	23.0 (4.0)	23.3 (3.5)	23.1 (3.3)	23.2 (1.8)	23.1 (3.3)
Mean maternal age, y (SD)	23.7 (4.2)	24.2 (4.5)	24.6 (4.8)	24.0 (4.3)	23.6 (4.6)	24.1 (4.8)	23.0 (3.6)	24.1 (4.8)
Household size >5	38 (43)	58 (36)	22 (43)	36 (34)	62 (43)	58 (51)	1 (33)	57 (51)
Mean gestational age at birth, wk (SD)	38.0 (1.7)	38.0 (1.8)	38.0 (1.8)	38.0 (1.9)	37.6 (1.7)	37.5 (1.8)	38.0 (1.0)	37.5 (1.8)
Mean WAZ at birth (SD)	–1.297 (0.851)	–1.287 (0.949)	–1.368 (0.927)	–1.249 (0.961)	–1.355 (0.902)	–1.339 (1.044)	–1.000 (0.573)	–1.348 (1.053)
WAZ > –1	34 (38)	73 (45)	22 (43)	51 (46)	46 (32)	44 (39)	2 (67)	42 (38)
WAZ –1 to –2	35 (39)	46 (29)	14 (28)	32 (29)	66 (46)	40 (35)	1 (33)	39 (35)
WAZ –2 to –3	18 (20)	34 (21)	14 (28)	20 (18)	24 (17)	20 (18)	0 (0)	20 (18)
WAZ < –3	2 (2)	8 (5)	1 (2)	7 (6)	8 (6)	9 (8)	0 (0)	0 (0)
Mean LAZ at birth (SD)	–0.979 (0.965)	–0.938 (0.906)	–0.838 (1.029)	–0.985 (0.845)	–0.828 (1.033)	–0.858 (1.189)	–0.597 (0.621)	–0.8065 (1.201)
LAZ > –1	43 (48)	83 (52)	28 (55)	55 (50)	85 (59)	66 (58)	2 (67)	64 (58)
LAZ –1 to –2	31 (35)	59 (37)	20 (39)	39 (36)	37 (26)	32 (28)	1 (33)	31 (28)
LAZ –2 to –3	15 (17)	17 (11)	2 (4)	15 (14)	20 (14)	10 (9)	0 (0)	10 (9)
LAZ < –3	0 (0)	2 (1)	1 (2)	1 (1)	2 (1)	6 (5)	0 (0)	6 (5)
Mean exclusive breastfeeding, d (SD)	118 (68)	113 (70)	110 (66)	115 (72)	70 (64)	58 (63)	128 (20)	56 (63)
Water source								
Municipal supply	89 (100)	160 (99)	51 (100)	109 (99)	0 (0)	0 (0)	0 (0)	0 (0)
Tube well	0 (0)	0 (0)	0 (0)	0 (0)	135 (94)	111 (97)	3 (100)	108 (97)
Treated water	74 (83)	114 (71)^f^	33 (65)^g^	81 (74)	30 (21)	21 (18)	1 (33)	20 (18)
Open drain near home	32 (36)	60 (37)	21 (41)	39 (36)	7 (5)	1 (1)	0 (0)	1 (1)

Data are presented as No. (%) unless otherwise indicated.

Abbreviations: BDT, Bangladeshi taka; BMI, body mass index; Crypto, cryptosporidiosis; IQR, interquartile range; LAZ, length-for-age *z* score; SD, standard deviation; WAZ, weight-for-age *z* score.

^a^Children having at least 1 *Cryptosporidium* infection (either diarrheal or subclinical) during the first 24 months of life were included in the “Any Crypto” group. All others were included in the “No Crypto” group.

^b^No significant differences when compared with “No Crypto” group from Mirpur by χ^2^ or Wilcoxon test unless otherwise indicated.

^c^Children having at least 1 *Cryptosporidium* infection were further divided into “Diarrheal Crypto” or “Subclinical Crypto” based upon phenotype of initial stool sample of infection (diarrheal sample or monthly surveillance).

^d^No significant differences when compared with “No Crypto” group from Mirzapur by χ^2^ or Wilcoxon test unless otherwise indicated.

^e^1000 BDT is equivalent to approximately 12 US dollars.

^f^ *P* = .044.

^g^ *P* = .023.

**Figure 1.**
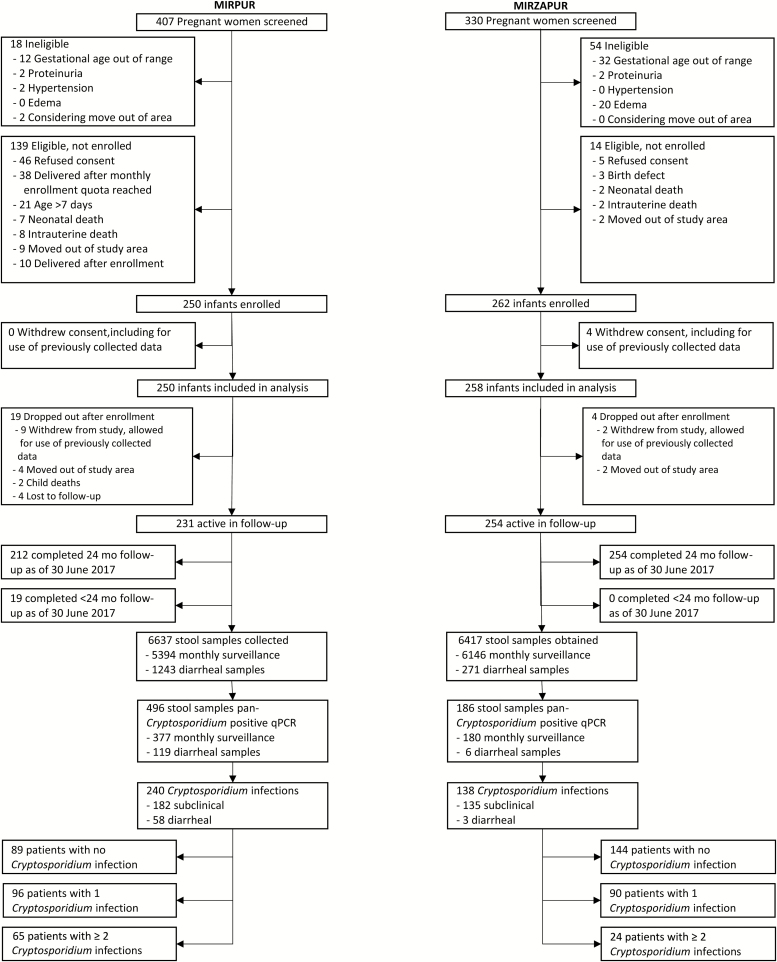
Study flow diagram. Abbreviation: qPCR, quantitative polymerase chain reaction.

Pregnant women in their second trimester were identified. In Mirpur, field research assistants performed a census. In Mirzapur, study participants were identified using a demographic surveillance system previously established by the Bangladeshi government [14]. Interested women meeting study criteria and providing informed consent underwent clinical examination, urinalysis, and gestational age confirmation by ultrasound. Exclusion criteria were age <18 years, gestational age ≥7 months, hypertension, edema, proteinuria, or intent to migrate from the study area (Figure 1). After delivery, infants assessed by the study medical officer (SMO) within the first 7 days of life were eligible for enrollment. For logistical reasons at Mirpur, the monthly maximum enrollment was 27.

The SMO collected demographic and socioeconomic data using a structured questionnaire at enrollment. Thereafter, field research assistants performed twice-weekly, in-home visits to interview caregivers and collect information regarding child morbidity and diarrhea. The SMO assessed all children monthly. Caregivers were encouraged to bring the child to the study clinic whenever the child developed symptoms of any illness, not only diarrhea. If acutely ill during an in-home visit, the child was referred to the study clinic. If the SMO determined that further treatment was needed, care was provided free of charge at the International Centre for Diarrhoeal Disease Research, Bangladesh (icddr,b) Mirpur Treatment Centre, the icddr,b Dhaka Hospital in Mirpur, or the Kumudini Hospital in Mirzapur.

Height and weight were measured for mothers at the infant enrollment visit. Infant length (to nearest 0.1 cm using length board and plastic tape) and weight (kilograms, measured with electronic scale; TANITA, HD-314) were obtained every 3 months. Weight-for-age *z* score (WAZ) and length-for-age *z* score (LAZ) were determined using World Health Organization Anthro software (version 3.2.2). The change in LAZ (Δ-LAZ) was calculated by subtracting the enrollment LAZ from that at 24 months.

### Detection of Cryptosporidiosis

Single, fresh stool specimens were collected from children every month (monthly surveillance) and during episodes of diarrhea. A modified Qiagen stool DNA extraction protocol was performed incorporating a 95°C incubation and a 3-minute glass bead–beating step (Qiagen, Valencia, California). All stool samples were tested for *Cryptosporidium* by quantitative polymerase chain reaction (qPCR) assay modified from Liu et al [15] (Supplementary Methods). A cycle threshold of 40 was used. The pan-*Cryptosporidium* primers and probes target the 18S gene in multiple species known to infect humans. All samples positive for *Cryptosporidium* were subsequently assessed for species identification using previously described Lib13 qPCR for *C. hominis* and *C. parvum* and a novel *C. meleagridis* qPCR assay [16, 17] (Supplementary Methods).

### Definitions

Diarrhea was defined as ≥3 loose stools within a 24-hour period as reported by the child’s caregiver. Infection with *Cryptosporidium* was defined as detection of *Cryptosporidium* DNA by qPCR from stool. Samples were grouped into a single infection if occurring within 65 days of a preceding positive sample based upon *gp60* genotyping of a subset of samples (≤65 days: 93% concordance; >65 days: 33% concordance) [18]. *Cryptosporidium* infection phenotype (diarrheal or subclinical) was based upon symptoms at the time of initial *Cryptosporidium*-positive stool sample (diarrheal stool vs monthly surveillance). Causative species was assigned based upon results of any stool sample obtained during an infection.

### Statistical Analysis

Analyses were performed using R version 3.3.3, 32-bit. The *P* values for Table 1 were calculated using Wilcoxon or χ^2^ tests. Differences in cumulative incidence of first *Cryptosporidium* infection, depicted by Kaplan-Meier curves, were assessed by log-rank test. Association of cryptosporidiosis during the first 24 months of life with Δ-LAZ was assessed using *t* test initially, and subsequently evaluated using stepwise linear regression to adjust for potentially important confounders. Variables considered in the regression with entry *P* value <.1: *Cryptosporidium* infection, enrollment LAZ, maternal body mass index (BMI), household income, water source, water treatment, and exclusive breastfeeding days. Association of recurrent *Cryptosporidium* infections with Δ-LAZ was evaluated among children with 0, 1, and ≥2 detected infections. Differences in Δ-LAZ among these groups were tested using 1-way analysis of variance (ANOVA) with post hoc analysis by Tukey correction for multiple comparisons, and further evaluated using stepwise linear regression adjusting for the same potential confounders stated above.

### Ethical Considerations

The Ethics and Research Review Committee at icddr,b approved this study; the Institutional Review Board of the University of Virginia provided a reliance agreement. Informed written consent was obtained from a parent or guardian of each child.

## RESULTS

### Cohort Characterization

Pregnant women in urban Mirpur (n = 407) and rural Mirzapur (n = 330) underwent clinical assessment, urinalysis, and ultrasonography in their second trimester (Figure 1). Eighteen women at Mirpur and 54 at Mirzapur were ineligible based on predefined exclusion criteria. Following delivery, 250 infants at Mirpur and 262 at Mirzapur were enrolled. Reasons for nonenrollment included parental or guardian refusal to consent, delivery after attainment of monthly or overall enrollment quota, enrollment assessment occurring >7 days after delivery, and intrauterine or neonatal death. After enrollment, 19 children at Mirpur and 8 at Mirzapur discontinued the study, primarily due to parental/caregiver withdrawal or migration. Therefore, 231 children from Mirpur and 254 from Mirzapur were actively followed. Through 30 June 2017, 212 and 254 children had completed 24 months of follow-up from Mirpur and Mirzapur, respectively. Data from children not yet completing 24 months of follow-up were included in all analyses except that of Δ-LAZ. Characteristics of enrolled children are summarized in Table 1 and stratified by absence or presence of at least 1 detected *Cryptosporidium* infection (either diarrheal or subclinical) during the surveillance period.

### Cryptosporidiosis Burden

From 1 July 2014 through 30 June 2017, 6637 and 6417 stool samples were collected in Mirpur and Mirzapur, respectively, and tested for *Cryptosporidium* by qPCR (Figure 1). Fewer diarrheal stools were collected in Mirzapur (n = 271) than Mirpur (n = 1243) despite high rates of stool collection during diarrheal episodes in both sites (99% and 85% in Mirzapur and Mirpur, respectively). In Mirpur, *Cryptosporidium* was detected in 496 separate stool samples representing 240 distinct infections among 161 children. Of these infections, 182 were subclinical (76%, initial detection in a monthly surveillance stool) and 58 were diarrheal (initial detection in a diarrheal stool sample). Of 250 children enrolled at Mirpur, 36% (n = 89) had no detectable *Cryptosporidium* infections, 38% (n = 96) had 1, and 26% (n = 65) had ≥2. In Mirzapur, *Cryptosporidium* was detected in 186 separate stool samples representing 138 distinct infections among 114 children. One hundred thirty-five infections were phenotypically subclinical (98%) and 3 were diarrheal. Of 258 children enrolled at Mirzapur, 56% (n = 144) had no detectable *Cryptosporidium* infections, 35% (n = 90) had 1, and 9% (n = 24) had ≥2. When stratified by site and according to presence or absence of detected *Cryptosporidium* infection during the first 2 years of life as well as whether *Cryptosporidium* infections were diarrheal or subclinical, the only statistically significant differences observed in baseline characteristics were for use of treated water in Mirpur. Compared with the no cryptosporidiosis group (n = 74 [83.2%]), use of treated water was less common among those with any cryptosporidiosis (n = 114 [70.8%], *P* = .044) and the subset with diarrheal cryptosporidiosis (n = 33 [64.7%], *P* = .023; Table 1). Boiling was the predominant form of water treatment in both sites.

**Figure 2.**
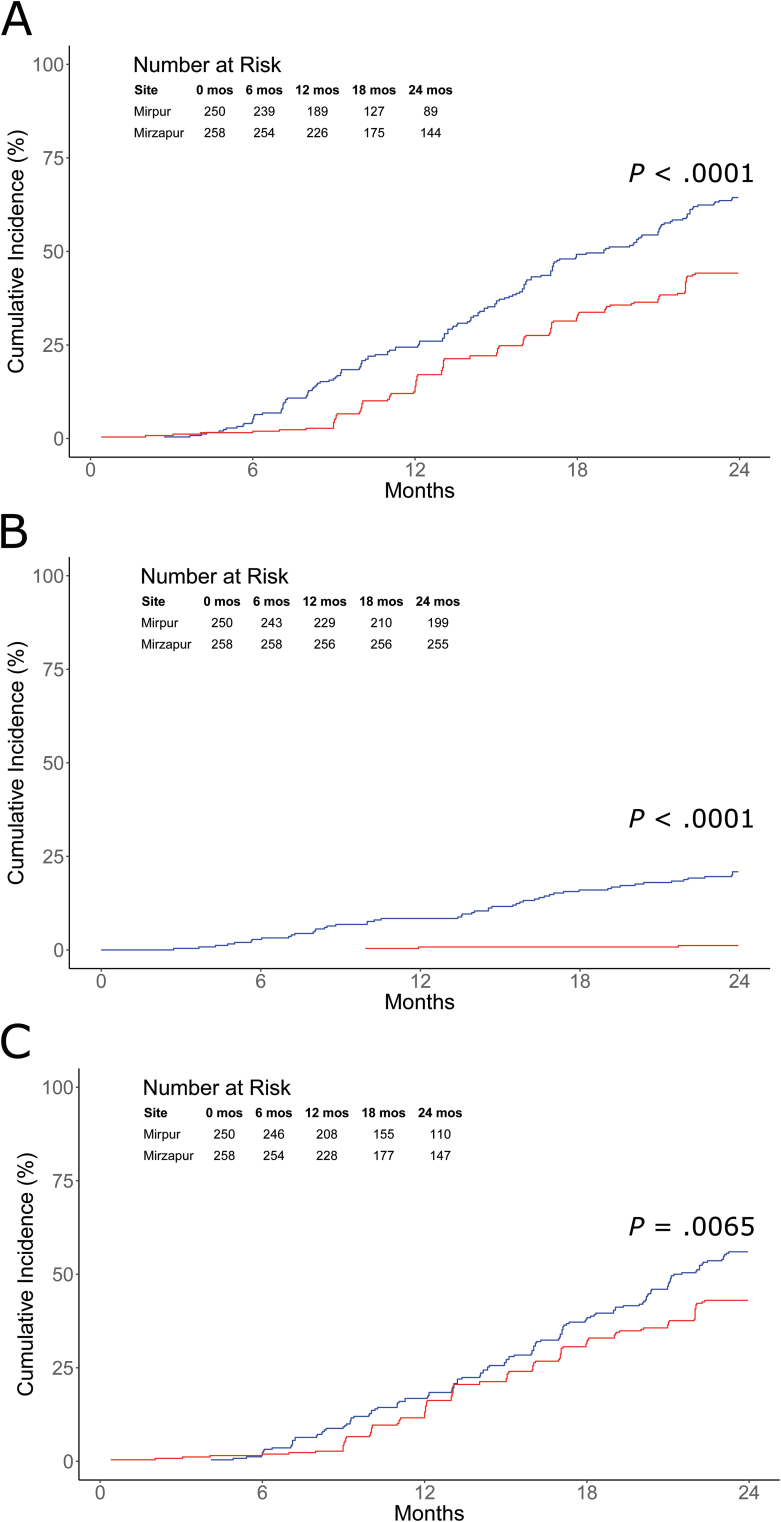
Cryptosporidiosis cumulative incidence curves by study site using Kaplan-Meier method for any (*A*), diarrheal (*B*), and subclinical (*C*) *Cryptosporidium* infections. Blue line = Mirpur; red line = Mirzapur.

Children in Mirpur experienced their first *Cryptosporidium* infection at a significantly younger age and had a greater overall cumulative incidence by 2 years compared with Mirzapur (Figure 2A–C). In Mirpur, the earliest *Cryptosporidium* infection was detected at 83 days of life; 61 (24%) children had at least 1 infection within the first year of life and 161 (64%) by age 2. Median age at first infection was 429 days (interquartile range [IQR], 275–547 days). Sixty-one diarrheal infections among 51 children occurred in Mirpur, with 22 occurring within the first year of life. In Mirzapur, the earliest *Cryptosporidium* infection was detected at 12 days of life; 32 (12%) had at least 1 infection within the first year of life and 114 (44%) by 2 years. Median age at first infection was 443 days (IQR, 337–550 days). Only 3 diarrheal infections were identified in Mirzapur, with 2 occurring within the first year of life.

### Cryptosporidiosis and Growth Faltering

Long-term impact on child growth was assessed using LAZ. Moderate or severe stunting (LAZ ≤ –2) at birth was found in 14% (34/250) and 15% (38/258) of children from Mirpur and Mirzapur, respectively. Mean LAZ consistently decreased at both sites with increased prevalence of stunting over the first 2 years of life (Supplementary Tables 2 and 3; Supplementary Figures 2 and 3). At 2 years, 33% (69/210) of children in Mirpur and 20% (51/254) in Mirzapur were moderately or severely stunted.

**Figure 3.**
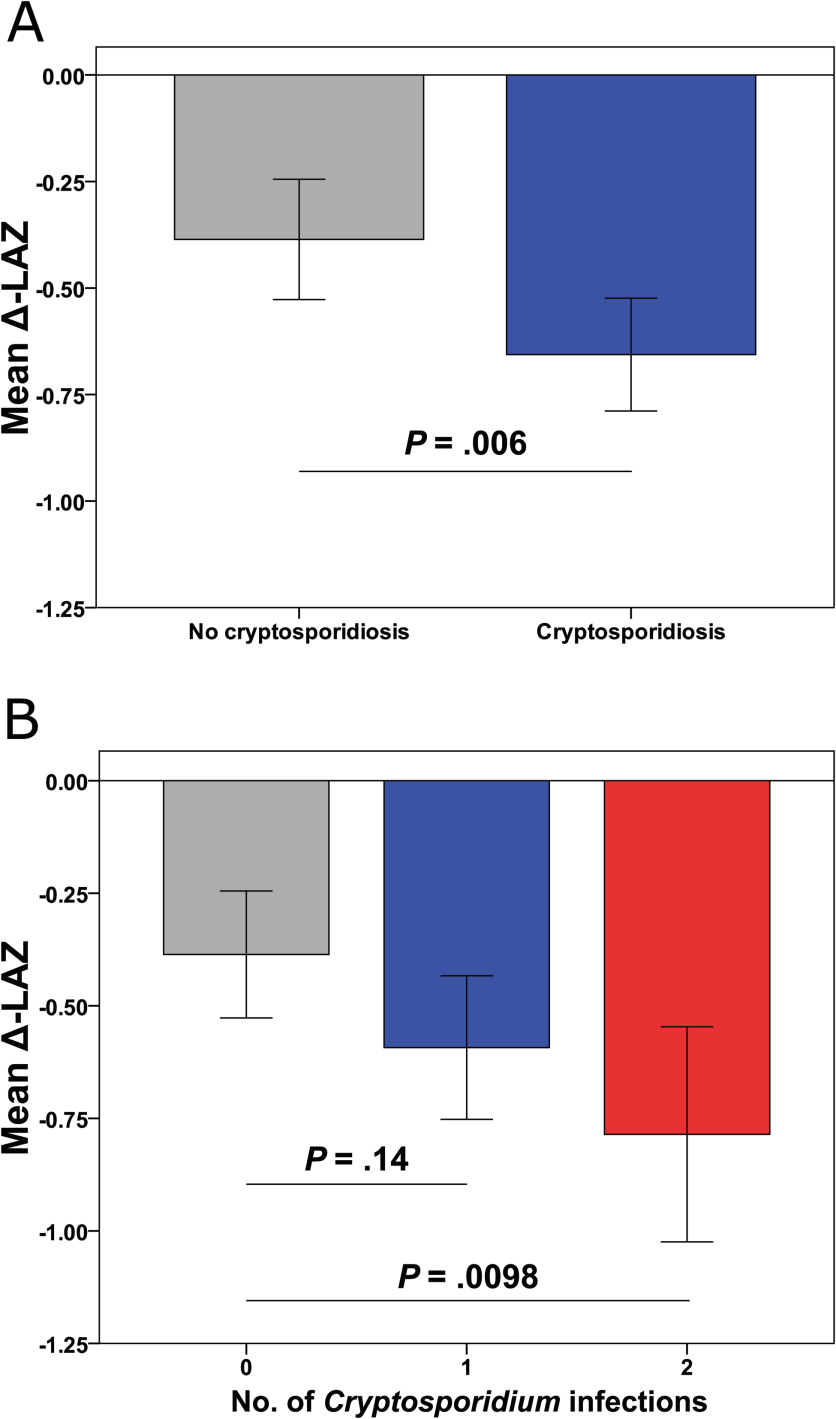
Association of cryptosporidiosis and unadjusted mean change in length-for-age *z* score at 24 months compared with enrollment (Δ-LAZ). *A*, Stratified into children with no cryptosporidiosis (n = 205) or any cryptosporidiosis (n = 259) detected during follow-up. *P* value by *t* test. *B*, Stratified into zero (n = 205), 1 (n = 174), or ≥2 (n = 85) episodes of cryptosporidiosis during follow-up. *P* values by post hoc Tukey multiple comparisons test following analysis of variance.

Delta-LAZ was calculated as the difference between LAZ at 24 months and that at enrollment. Including all participants at both sites, mean Δ-LAZ for children with no detected *Cryptosporidium* infections was –0.386 (n = 205) vs –0.656 (n = 259) in children with at least 1 infection (*P* = .0062; Figure 3A). Adjusting for confounding variables (enrollment LAZ, maternal BMI, household size, household income, water source, water treatment, and exclusive breastfeeding days), cryptosporidiosis during the first 2 years of life was significantly associated with an absolute decline in Δ-LAZ of 0.215 (*P* = .0088; Table 2). Site-specific analysis demonstrated that cryptosporidiosis was associated with a statistically significant decrease in adjusted Δ-LAZ at Mirzapur (–0.253, *P* = .011), but not at Mirpur (–0.213, *P* = .13).

**Table 2.** Regression Analysis and Parameter Estimates of Association of Cryptosporidiosis With Δ-Length-for-Age *z* Scores at 2 Years

Parameter	Parameter Estimate	*P* Value
Cryptosporidiosis	–0.215	.0088
LAZ at enrollment	–0.606	<.0001
Maternal BMI	0.035	.0016
Household size	–0.020	.2833
Household income (per 1000 BDT)	0.0065	.0156
Municipal source of water	–0.220	.4147
Tube well source of water	–0.043	.8714
Water source for feeding	0.321	.2826
Treatment of water	0.146	.1222
Exclusive breastfeeding days	–0.0015	.0109

Abbreviations: BDT, Bangladeshi taka; BMI, body mass index; LAZ, length-for-age *z* score.

Mean Δ-LAZ was significantly different by ANOVA among children with 0, 1, or ≥2 *Cryptosporidium* infections (*P* = .0095). Post hoc analysis demonstrated that, compared with those with no episodes of cryptosporidiosis (n = 205, –0.386), mean Δ-LAZ trended lower for children with 1 episode of cryptosporidiosis (n = 174, –0.593, *P* = .14) and reached statistical significance for those with ≥2 episodes (n = 85, –0.786, *P* = .0098; Figure 3B). After covariate adjustment, children with ≥2 episodes of cryptosporidiosis had a greater decrease in Δ-LAZ (–0.2385, *P* = .039) than those with 1 episode (–0.2056, *P* = .020; Supplementary Table 3).

### *Cryptosporidium* Species

Of 682 stools positive by pan-*Cryptosporidium* qPCR, 676 (representing 375 infections) were available for qPCR species testing. Species could not be determined for 107 infections (28%), likely due to lower sensitivity of the species-specific assay (Supplementary Methods; Supplementary Figure 1). *Cryptosporidium* species was determined for 166 and 102 infections for Mirpur and Mirzapur, respectively. Overall, *C. hominis* (total n = 148; 35 diarrheal) and *C. meleagridis* (total n = 100; 4 diarrheal) monoinfections were most common. Multispecies coinfections were infrequent, with 13 (5 diarrheal) *C. hominis/C. meleagridis*, 1 (diarrheal) *C. hominis/C. parvum*, and 1 (subclinical) *C. parvum/C. meleagridis* coinfections.

**Figure 4.**
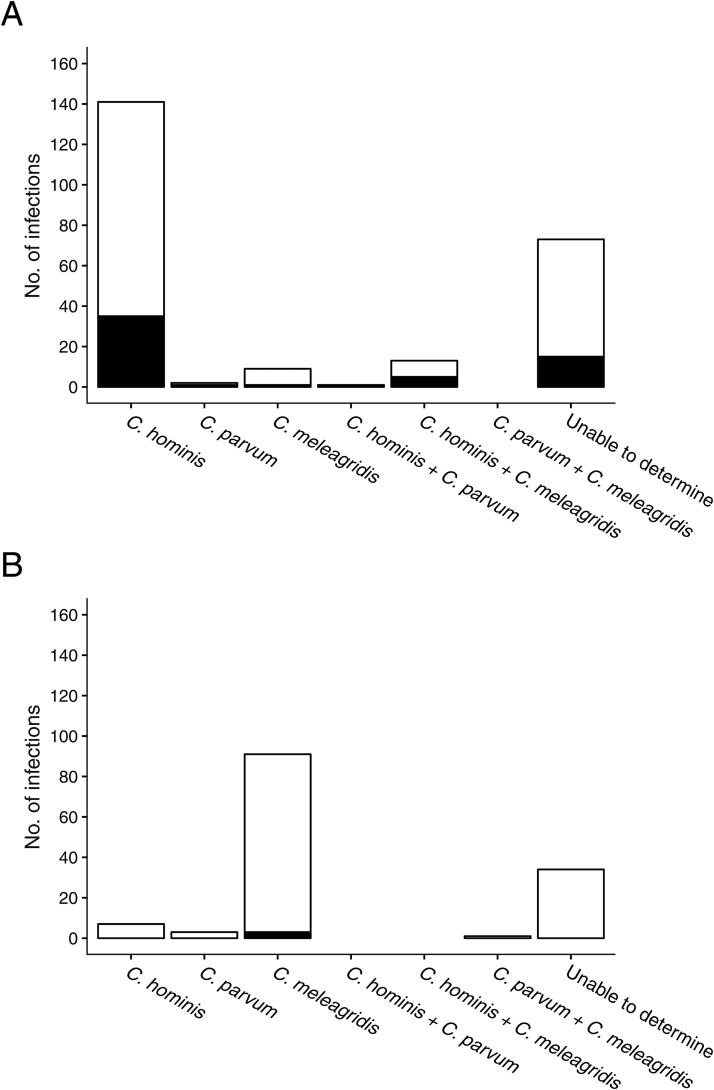
*Cryptosporidium* species detected and infection phenotype at urban Mirpur (*A*) and rural Mirzapur (*B*) sites. Solid bar indicates the number of diarrheal infections and open bar indicates subclinical infections.

A marked difference in predominant *Cryptosporidium* species was observed between study sites. In urban Mirpur, *C. hominis* was identified in 93% of infections in which species could be determined (n = 155, 141 monoinfections; Figure 4A). Forty-one infections in which *C. hominis* was detected were diarrheal and 114 were subclinical. *Cryptosporidium meleagridis* was the second most frequently identified species occurring in 13% (n = 22, 9 monoinfections), and *C. parvum* was identified in 2% (n = 3, 2 monoinfections). In rural Mirzapur, the predominant species was *C. meleagridis* (90%; n = 92, 91 monoinfections; Figure 4B), while *C. hominis* was identified in 7% (n = 7, all monoinfections) and *C. parvum* in 4% (total n = 4; 3 monoinfections). Monoinfections with *C. meleagridis* were nearly all asymptomatic (96%; subclinical, n = 96; diarrheal, n = 4). Of 9 diarrheal infections in which *C. meleagridis* was identified, 5 were coinfections with *C. hominis*.

## DISCUSSION

We established a longitudinal birth cohort in Bangladesh comprised of 1 rural and 1 urban location to better understand the natural history of cryptosporidiosis in disparate settings. Intensive, active surveillance revealed a high burden of cryptosporidiosis with 64% and 44% of children at the urban and rural site, respectively, experiencing at least 1 *Cryptosporidium* infection by age 2 years. Consistent with similar prior active surveillance cohort studies in diverse geographic locations, most *Cryptosporidium* infections occurred in the absence of diarrhea, which we term subclinical [2, 3, 7, 9, 19]. The predominance of subclinical infection was particularly striking in rural Mirzapur where only 2% of cryptosporidiosis episodes were diarrheal. Furthermore, a marked difference between the 2 sites in the predominant *Cryptosporidium* species was observed, with *C. hominis* most common in the urban site and *C. meleagridis* in the rural site. Despite these differences, when the cohort was analyzed as a whole, any cryptosporidiosis during the first 2 years of life was associated with a significant decrement in growth attainment, consistent with prior studies [7, 10, 11]. This adverse association was greatest in children with ≥2 episodes of cryptosporidiosis. When considered separately, the magnitude of effect on Δ-LAZ was similar at both sites, though reaching statistical significance only in the rural site; this finding is particularly striking as nearly all *Cryptosporidium* infections were subclinical in this location. Collectively, these results suggest that, even without overt diarrhea, cryptosporidiosis is associated with subsequent impaired growth.

The difference in predominant *Cryptosporidium* species observed between the sites is striking and suggests setting-specific modes of exposure. Our finding that *C. meleagridis* predominated in Mirzapur contrasts with prior work showing *C. hominis* as the most common species in this rural site; however, that study evaluated stool collected during moderate to severe diarrhea [20]. *Cryptosporidium meleagridis* as a cause of medically attended diarrhea has been described in diverse geographic regions including Peru, China, and Cambodia, where it comprised 23% of *Cryptosporidium*-positive stools [21–24]. To our knowledge, our data from rural Bangladesh is the first description of *C. meleagridis* as a major species infecting children in the absence of diarrhea, yet potentially associated with a shortfall in child growth. This suggests a heretofore unrecognized burden of subclinical cryptosporidiosis attributable to a species previously considered an uncommon human pathogen. This finding has significant implications for interventions aimed at reducing *Cryptosporidium*-attributable morbidity. Vaccines targeting *C. hominis* may reduce overt diarrheal cryptosporidiosis yet be ineffective in prevention of *Cryptosporidium*-associated developmental faltering if subclinical disease due to other species persists.

A limitation inherent to the observational design is an inability to fully account for all potentially confounding variables. Notably, testing for symptomatic or subclinical presence of enteric pathogens other than *Cryptosporidium* was not performed. We cannot determine whether and to what extent enteropathogens other than *Cryptosporidium* are contributing to growth impairment in this cohort, nor whether coinfections may result in additive or synergistic effect. It is possible that detection of *Cryptosporidium* is simply a surrogate indicator of some other exposure and therefore associated with, but not directly causative of, the observed growth impairment. This alternative explanation could be supported by the observation that water treatment was less common in the urban site among children who experienced cryptosporidiosis. Though statistically significant, the magnitude of difference in water treatment (83% vs 71%) seems insufficient to account for the observed differences in growth. Moreover, in the rural site where the association between cryptosporidiosis and impaired growth attainment was greatest, water treatment was infrequent, with no differences observed between children with or without cryptosporidiosis.

Additional study-specific limitations include the definitions used to delimit infections and the characterization of each infection as diarrheal or subclinical. *Cryptosporidium* infections may be prolonged with oocysts shed intermittently and often in the absence of diarrheal symptoms [25, 26]. This, coupled with practical limitation in frequency of surveillance stool collection and use of species-level identification, precluded exact determination of when 1 infection ceased and another began. We chose to define distinct infections when >65 days separated stools with detectable *Cryptosporidium* based upon concordance of *gp60* genotypes performed on a subset of samples. This definition could introduce bias in either direction by over- or undercounting the number of distinct infections. However, most distinct infections using this definition were separated by many months; therefore, the potential is greater for misclassifying multiple infections as a single infection, thereby underestimating the true frequency of cryptosporidiosis. Infections were classified as diarrheal or subclinical based on presence or absence of symptoms at the time of first *Cryptosporidium* detection. Sole use of the first timepoint of *Cryptosporidium* detection may bias toward overclassification of infections as subclinical, thereby underestimating somewhat the burden of diarrheal cryptosporidiosis.

Based upon our findings, we again suggest that not only diarrhea but also child growth may be considered an important clinical outcome of cryptosporidiosis. Future studies should aim to further characterize the ecology and prevalence of *Cryptosporidium* species, recognizing that some may infect without overt diarrhea yet still may impair growth. Further characterization of modes of transmission, which may differ by local environment and predominant species, must inform strategies to interrupt transmission. Our findings also support renewed efforts to better understand human immune responses to *Cryptosporidium* and elucidation of immunologic correlates of protection. It is only through such concerted and multidisciplinary efforts that cryptosporidiosis and its associated long-lasting sequelae may be abated.

## Supplementary Data

Supplementary materials are available at *Clinical Infectious Diseases* online. Consisting of data provided by the authors to benefit the reader, the posted materials are not copyedited and are the sole responsibility of the authors, so questions or comments should be addressed to the corresponding author.

Supplementary Data

## Notes

***Author contributions.*** W. A. P., R. H., and A. S. G. F. conceived the study. Field work, data gathering, and laboratory studies at icddr,b were performed by S. A., E. A., M. A., M. K., and F. T., with T. A., R. H., and A. S. G. F. providing supervision. C. B. and C. A. G. performed the species polymerase chain reaction. K. L. S., H. C., and J. Z. M. performed statistical analyses. K. L. S. wrote the manuscript with input from C. A. G., P. S. K., H. C., J. Z. M., and W. A. P. All authors contributed to revisions and approved the final version.

***Acknowledgments.*** We express our deepest appreciation to the children and parents from Mirpur and Mirzapur for participating in this study.

***Financial support.*** This study was supported by the National Institutes of Health (grant numbers R01 AI043596 to W. A. P. and T32 AI055432 to K. L. S.) and the Bill & Melinda Gates Foundation (grant number OPP1100514 to A. S. G. F.).

***Potential conflicts of interest.*** All authors: No reported conflicts. All authors have submitted the ICMJE Form for Disclosure of Potential Conflicts of Interest. Conflicts that the editors consider relevant to the content of the manuscript have been disclosed.
