# Supplementary material for: Species of Cryptosporidia Causing Subclinical Infection Associated With Growth Faltering in Rural and Urban Bangladesh: A Birth Cohort Study
Source: Clin Infect Dis. 2018 Apr 20;67(9):1347–55. doi: 10.1093/cid/ciy310 (PMC6186860; doi:10.1093/cid/ciy310)
Supplement: Supplementary Data [file ciy310_suppl_supplementary_data.docx]

**Supplemental Methods**

*Study site descriptions*

Mirpur is a densely populated, urban neighborhood with an average of 5.5 people living in 1.6 rooms for participants in this study. Annual median household income of participants was 12,950 (IQR 10,000-20,000) Bangladeshi Taka (approximately $158 USD, Table 1). Household water source was primarily via the municipal system. 74% of households boiled their water prior to use while 25% did not treat their water in any manner. More than 98% of households utilized a septic tank/toilet or ventilated or water-sealed latrines; however, open sewers flow throughout the neighborhood, frequently located directly adjacent to participant homes.

Mirzapur is a rural subdistrict northwest of Dhaka. On average, six household members occupy 2.7 rooms. Annual median household income of participants was 15,000 (IQR 10,000-26,000) Bangladeshi Taka (approximately $182 USD). Household water source was primarily tube well and 80% of households did not perform any water treatment prior to use while 16% boiled their water. Half of households utilize improved sanitation via ventilated or water-sealed latrines.

*Modifications to qPCR for detection of Cryptosporidium, E. histolytica, and Giardia*

The fluorophore Texas Red was used for the pan-*Cryptosporidium* probe, FAM for *E. histolytica*, and a MGB VIC probe was used to detect *Giardia* [1].

*C. meleagridis qPCR*

A novel *C. meleagridis* qPCR was developed using the probe and reverse primer of the pan-*Cryptosporidium* qPCR assay but incorporating a forward primer based on the sequence recognized by the *C. meleagridis* Scorpion probe as previously described (Cm_F: GCGAAAAAACCTGACTTAATGGAAA) [2,3]. Assay specificity was increased by the incorporation of the non-extendable primer blocking 3’ spacer C3 oligonucleotides into the reaction (Ch_blck: GCGAAAAAACTCGACTTTATGGAAG/3SpC3/; Cp_blck: CGAAAAAACCTAACTTTATGGAAA/3SpC3/).

*Cryptosporidium species-specific qPCR and validation*

Assay performance and specificity was initially optimized using DNA extracted from fecal material spiked with either *C. parvum* oocysts (Waterborne Inc.) or *C. hominis* and *C. meleagridis* amplicons prior to DNA purification (Fig 1) [4]. The assay was then validated using a panel of clinical specimens which were identified as pan-*Cryptosporidium* positive by qPCR followed by amplification and sequencing of a divergent region within the *Cryptosporidium* 18S rRNA gene to ascertain whether they were *C. hominis*, *C. meleagridis* or *C. parvum.* The final assay conditions consisted of primer and blocking oligonucleotides 0.4 μM; probe 0.2μM; in 1x Bio-Rad IQ Powermix. Initial DNA denaturation and Taq activation incubation (95^o^C for 3 min), followed by 40 repeat cycles (95^o^C for 10sec, 60^o^C for 1 min) to amplify the target DNA. Amplification was monitored by tracking the cleavage of Texas-Red fluorophore in the genus specific probe from its quencher (Bio-Rad CFX96 instrument).

This assay preferentially detected *C. meleagridis* however it also detected the closely related species of *C. hominis* and *C. parvum.* For this reason all samples were analyzed by both the *C. meleagridis* assay and the LIB13 *C. hominis* and *C. parvum* assays which do not detect *C. meleagridis* [2]. There was a 10 cycle difference between equivalent amounts of *C. meleagridis* and *C. hominis* (which equates to a >10^3^ fold change in target concentration). Between *C. meleagridis* and *C. parvum* there was a greater than 20 cycle difference which equates to a >10^6^ fold change. The amplification efficiencies of the LIB13 and the *C meleagridis* assays were not significantly different therefore a ΔCq < 8 cycle cut off was applied to the identification of *C. meleagridis* and *C. hominis* co-infections. The pan-*Cryptosporidium* positive assay was more sensitive than the species specific assay (Lib13 *C.hominis* ΔqPCR: 7.002 + 0.6325 n=100; *C. meleagridis* ΔqPCR: 5.616 + 1.051; n=112 p<0.0001) although the qPCR assays efficiency was not significantly different (pooled slope =0.96).

**Supplemental Figure 1:** A) Limit of *Cryptosporidium meleagridis* qPCR assay detection. Cq value is shown on the x-axis and on the y-axis the amplicon DNA concentration. B) Comparison in study samples of species specific assays sensitivity (Cq values shown on the y-axis) with that of the pan-Cryptosporidium diagnostic assay (Cq values on x-axis)


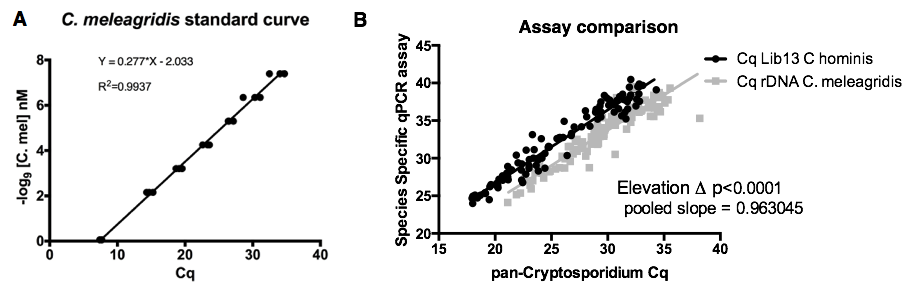


**Supplemental Figure 2: Mean length-for-age adjusted z-score over first 24 months of life by site.** Mirpur (urban), blue; Mirzapur (rural), red. Error bars indicate 95% confidence interval. Horizontal line indicates W.H.O. reference population mean.


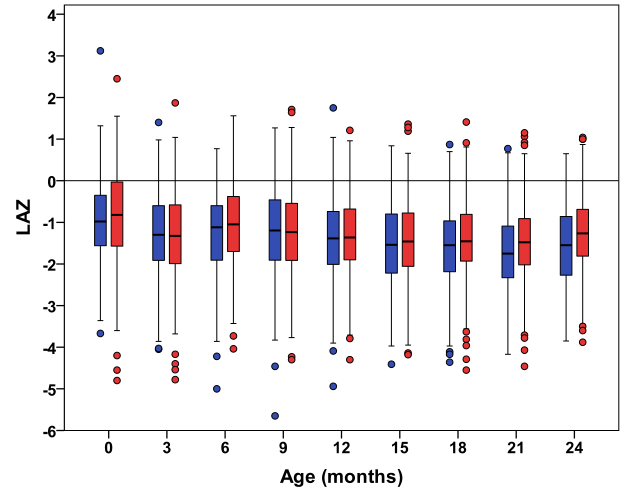


**Supplemental Table 1:** Prevalence by severity of stunting in urban Mirpur over first two years of life by length-for-age z-score (LAZ).

|  | Enrollment (n=250) | 3 months (n=232) | 6 months (n=225) | 9 months (n=226) | 12 months (n=230) | 15 months (n=219) | 18 months (n=219) | 21 months (n=205) | 24 months (n=210) |
| --- | --- | --- | --- | --- | --- | --- | --- | --- | --- |
| Not stunted^a^ n (%) | 126 (50.4%) | 92 (39.7%) | 103 (45.8%) | 90 (39.8%) | 76 (33.0%) | 66 (30.1%) | 56 (25.6%) | 46 (22.4%) | 58 (27.6%) |
| Mild stunting^b^ n(%) | 90 (36%) | 88 (37.9%) | 75 (33.3%) | 87 (38.5%) | 94 (40.9%) | 88 (40.2%) | 95 (43.4%) | 77 (37.6%) | 83 (39.5%) |
| Moderate stunting^c^ n(%) | 32 (12.8%) | 40 (17.2%) | 41 (18.2%) | 39 (17.3%) | 46 (20.0%) | 49 (22.4%) | 49 (22.4%) | 66 (32.2%) | 49 (23.3%) |
| Severe stunting^d^ n(%) | 2 (0.8%) | 12 (5.2) | 6 (2.7%) | 10 (4.4%) | 14 (6.1%) | 16 (7.3%) | 19 (8.7%) | 16 (7.8%) | 20 (9.5%) |

^a^ LAZ > -1

^b^ LAZ -1 to -2

^c^ LAZ -2 to -3

^d^ LAZ <-3

**Supplemental Table 2:** Prevalence by severity of stunting in rural Mirzapur over first two years of life by length-for-age z-score (LAZ).

|  | Enrollment (n=258) | 3 months (n=258) | 6 months (n=258) | 9 months (n=256) | 12 months (n=256) | 15 months (n=255) | 18 months (n=254) | 21 months (n=254) | 24 months (n=254) |
| --- | --- | --- | --- | --- | --- | --- | --- | --- | --- |
| Not stunted^a^ n (%) | 151 (58.5%) | 106 (41.1%) | 121 (46.9%) | 99 (38.7%) | 83 (32.4%) | 80 (31.4%) | 71 (28%) | 71 (28%) | 93 (36.6%) |
| Mild stunting^b^ n(%) | 69 (26.7%) | 88 (34.1%) | 95 (36.8%) | 98 (38.3%) | 119 (46.5%) | 110 (43.1%) | 123 (48.4%) | 118 (46.5%) | 110 (43.3%) |
| Moderate stunting^c^ n(%) | 30 (11.6%) | 48 (18.6%) | 35 (13.6%) | 49 (19.1%) | 39 (15.2%) | 50 (19.6%) | 46 (18.1%) | 46 (18.1%) | 37 (14.6%) |
| Severe stunting^d^ n(%) | 8 (3.1%) | 16 (6.2%) | 7 (2.7%) | 10 (3.9%) | 15 (5.9%) | 15 (5.9%) | 14 (5.5%) | 19 (7.5%) | 14 (5.5%) |

^a^ LAZ > -1

^b^ LAZ -1 to -2

^c^ LAZ -2 to -3

^d^ LAZ <-3

**Supplemental Figure 3:** Prevalence and severity of stunting by age. A) Mirpur. B) Mirzapur. Orange = no stunting (LAZ greater than -1), gray = mild stunting (LAZ between -1 and -2), blue = moderate stunting (LAZ between -2 and -3), white = severe stunting (LAZ less than -3).

| **A**  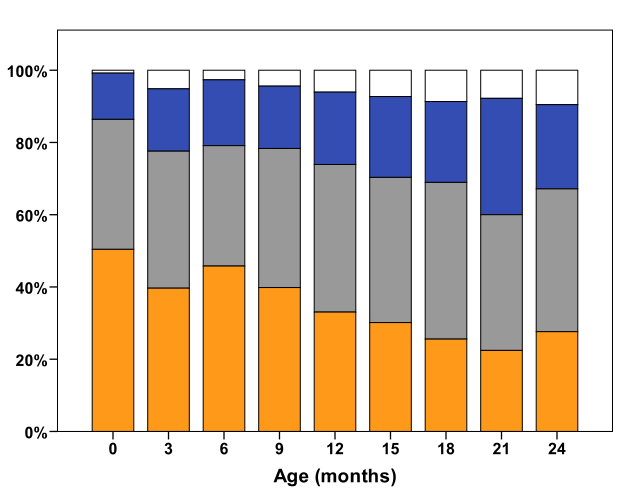 |
| --- |
| **B**  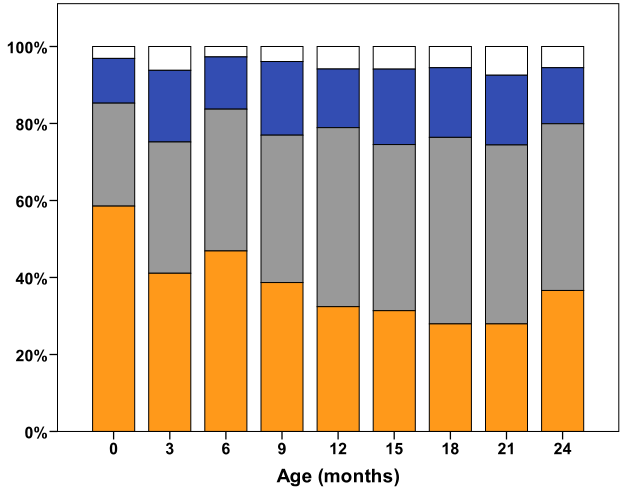 |

Supplemental table 3: Stepwise regression analysis by number of cryptosporidiosis episodes

| Variable | Parameter Estimate | p value |
| --- | --- | --- |
| Intercept | -1.9034 | <.0001 |
| 1 cryptosporidium infection | -0.2056 | 0.0198 |
| 2 or more cryptosporidium infections | -0.2385 | 0.0385 |
| LAZ_at enrollment | -0.6053 | <.0001 |
| Maternal BMI | 0.0353 | 0.0017 |
| Household_size | -0.0200 | 0.2860 |
| Household income (per 1000 BDT) | 0.0064 | 0.0167 |
| Municipal source of water | -0.2203 | 0.4152 |
| Tube well source of water | -0.0482 | 0.8556 |
| Water source for feeding | 0.3240 | 0.2786 |
| Treatment of water | 0.1442 | 0.1287 |
| Exclusive breast feeding days | -0.0016 | 0.0106 |

**Supplemental References**

1. Liu J, Kabir F, Manneh J, et al. Development and assessment of molecular diagnostic tests for 15 enteropathogens causing childhood diarrhoea: a multicentre study. Lancet Infect. Dis. **2014**; 14:716–724.

2. Hadfield SJ, Robinson G, Elwin K, Chalmers RM. Detection and Differentiation of Cryptosporidium spp. in Human Clinical Samples by Use of Real-Time PCR. J. Clin. Microbiol. **2011**; 49:918–924.

3. Stroup SE, Roy S, Mchele J, et al. Real-time PCR detection and speciation of Cryptosporidium infection using Scorpion probes. J. Med. Microbiol. **2006**; 55:1217–1222.

4. Liu J, Platts-Mills JA, Juma J, et al. Use of quantitative molecular diagnostic methods to identify causes of diarrhoea in children: a reanalysis of the GEMS case-control study. Lancet Lond. Engl. **2016**; 388:1291–1301.
